# Feasibility of externalized peritoneovenous shunt (EPVS) for malignant ascites

**DOI:** 10.1186/1477-7819-9-82

**Published:** 2011-07-21

**Authors:** Hiroyuki Tokue, Yoshito Takeuchi, Yasuaki Arai, Keitaro Sofue, Noriaki Sakamoto, Yoshito Tsushima, Keigo Endo

**Affiliations:** 1 Department of Diagnostic and Interventional Radiology, Gunma University Hospital, Maebashi, Gunma, Japan; 2 Division of Diagnostic Radiology, National Cancer Center Hospital, Tokyo, Japan

**Keywords:** Denver shunt, peritoneovenous shunt (PVS), externalized peritoneovenous shunt (EPVS), malignant ascites, palliative therapy

## Abstract

**Purpose:**

To evaluate a new modified peritoneovenous shunt therapy, the externalized peritoneovenous shunt (EPVS) system placement, used to treat patients with malignant ascites.

**Methods:**

We retrospectively reviewed 10 patients, who were not suited for conventional peritoneovenous shunts (PVS), with malignant ascites, which was refractory to medical therapies. Patient characteristics, technical success, efficacy, duration of EPVS placement, adverse events, and outcome were evaluated. Clinical efficacy of the EPVS was evaluated by the change in subjective symptoms.

**Results:**

The primary reasons for applying EPVS were severe anasarca in 4 patients, potential PVS dysfunction in 3 patients, poor performance status in 2 patients, and a history of PVS occlusion in 1 patient. EPVS was successfully placed in all patients, and it provided clinical efficacy in 8 patients (80%). Early death occurred within 7 days after EPVS placement in 2 patients because of renal failure. The median duration of EPVS placement was 10.4 days (range, 2-28 days). In 6 patients (60%), the EPVS was exchanged to conventional PVS sequentially, since the initial EPVS placement resulted in an improvement of the subjective symptoms of the patients, without serious complications.

**Conclusion:**

EPVS placement may be an option for patients with malignant ascites who may not be appropriate for conventional PVS placement.

## Background

A peritoneovenous shunt (PVS), which is also known as a Denver shunt, may be effective for palliating symptoms in patients with malignant ascites, which contribute to a deterioration of the patient's quality of life (QOL) and which are refractory to conservative nonsurgical therapies. Various shunts have been designed to use as peritoneovenous shunting [1,2], and radiological insertion of the Denver shunt may be the most widely used technique for nonsurgical PVS implantation in our country. However, the mortality rate of PVS implantation has been reported to be rather high, and indications are limited [3-6]. Possible contraindications for PVS implantation include ascites that is infected, hemorrhagic, chylous, or with loculated malignant effusion, advanced cardiac or renal failure, elevated serum bilirubin levels (6 mg/dL), portal hypertension, massive pleural effusion, coagulation disorders, and poor performance status (PS).

In addition, since a long subcutaneous tunnel should be constructed in the implantation of a PVS, a PVS once implanted in the subcutaneous tissue cannot be readily modified, even when the system may get infected or occluded [7], particularly in patients with severe anasarca. Furthermore, postoperative incisional separation of wound is considered to be main inappropriate reason for PVS in patients with sever anasarca.

We applied an externalized peritoneovenous shunt (EPVS) in 10 patients in whom conventional PVS was considered to be inappropriate due to various reasons and evaluated the feasibility of this new alternative method. And EPVS was inserted to estimate the patients whether they can tolerate subsequent PVS placement.

## Patients and methods

We retrospectively reviewed 10 patients with refractory malignant ascites who underwent EPVS placement between January 2005 and December 2010. There were 32 cases of conventional PVS during the same period. Inclusion criteria of EPVS were as follows: (1) malignant ascites was confirmed cytologically or clinically, (2) ascites was refractory to conservative nonsurgical therapies, (3) there was no evidence of infection of the ascites, (4) QOL was deteriorated because of the ascites, and (5) conventional PVS was considered to inappropriate because of severe anasarca, potential PVS dysfunction, history of PVS occlusion or poor PS.

Potential PVS dysfunction was defined as possible shunt dysfunction due to particular characteristics of ascites such as bloody or chylous. Poor PS was defined as a life expectancy that was considered to be less than a month. These patients did not have liver dysfunction, portal hypertension, massive pleural effusion, or coagulation disorders. Refractory ascites was diagnosed when the ascites failed to respond to conservative therapy (fluid restriction to 1000 mL/day, 100 mg/day of spironolactone, or 40 mg/day of furosemide for 4 weeks), or when the patients had intolerance to these conservative therapies because of azotemia [8-10].

**Figure 1:**
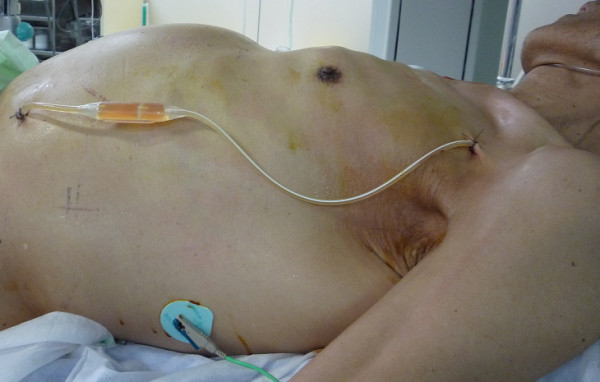
**A photograph of EPVS placement in a patient with chondrosarcoma (Patient No. 10)**. The venous and peritoneal parts of the Denver shunt catheter have been inserted, and the catheter is on the skin. (The patient provided consent for the photograph to be published.)

We employed a PVS kit (Denver-PAK; Denver Biomaterials, Inc., Golden, CO, USA) in all patients. Procedures were carried out under local anesthesia by interventional radiologists. The abdominal catheter was inserted into the Douglas cavity through the 16-F sheath. Then the venous catheter was inserted into the right or the left subclavian vein and was placed at the lower portion of the superior vena cava through the 12-F sheath. Ultrasound and X-ray fluoroscopy was used for the guidance of the catheters insertion (Figure 1).

Technical success was defined as the creation of the shunt and catheter insertions into both the abdominal cavity and superior vena cava. Immediately after the procedures, 500 mg of hydrocortisone sodium succinate and 20 mg of furosemide were administered intravenously to reduce acute biochemical reactions related to the shunting and strain on hemodynamics. For the patients with normal renal function (n = 8), 2 g/day of cefazolin (CEZ) was given intravenously for 3 days. In all patients (n = 10), 3 μg/kg/min of catecholamines was administered in order to maintain more than 1000 ml/day of urine volume for 3 days. In the patients who had less than 10 × 10^3 ^cells/mm^3 ^of blood platelets (n = 2), 100 mg/day of gabexate was administered for 7 days. Central venous pressure was continuously monitored in order to minimize fluid overload, and chest X-ray pictures were checked in order to detect pulmonary edema.

Patient characteristics, technical success, efficacy, the duration of EPVS placement, adverse events, and outcome were retrospectively evaluated. Technical success was defined as the creation of the shunt with catheter insertions into both the abdominal cavity and the superior vena cava. We also evaluated hematological parameters, abdominal girth, and diuresis before and after EPVS placement. Improvement of anasarca was evaluated as a decrease of abdominal girth.

We asked patients about subjective symptoms before and after the EPVS procedures. Clinical efficacy of the EPVS was evaluated by the change in subjective symptoms. Each EPVS procedure was classified into 2 groups: effective, in which the duration of the improvement of at least one of 3 subjective symptoms from the ascites (abdominal distention, anorexia, and nausea/vomiting) was 7 days or more; ineffective, in which there was no subjective improvement, or the duration of symptom improvement was less than 7 days. If EPVS was exchanged to PVS within 7 days, EPVS was judged effective when at least one of 3 subjective symptoms from the ascites was improved before EPVS was exchanged to PVS.

PS was evaluated by the Eastern Cooperative Oncology Group Performance Status (ECOGPS) [11]. Adverse events (AEs) were categorized according to the clinical practice guidelines of the Society of Interventional Radiology. Major AEs were defined as those necessitating an increased level of care, major therapy, or prolonged hospitalization, and those resulting in permanent sequelae or death. Minor AEs were defined as those necessitating nominal therapy or observation only [12].

When conventional PVS therapy was followed by EPVS implantation, a subcutaneous tunnel was created first, and the center part of the device was placed. The abdominal catheter was exchanged by injecting saline solution into the abdominal cavity in order to prevent damage to the adjacent organs. The venous side catheter was also exchanged using a guidewire.

Informed consent was obtained from each patient before EPVS implantation was performed, and the institutional review board of our institution approved this technique. This study was conducted in accordance with the amended Helsinki Declaration.

## Results

Characteristics of the 10 patients (3 men and 7 women; mean (SD) age, 52 (13) years; range, 22-69) are listed in Table 1. The reasons for the use of EPVS placement instead of a conventional PVS were poor PS in 4 patients, severe anasarca in 4 patients, potential PVS dysfunction in 4 patients, and a history of PVS occlusion in 2 patients (with overlap). The patency period of initial PVS in the patients (No. 4 and No. 8) who had a history of PVS occlusion was 192 and 69 days. Subsequent PVS placement might be possible because the function of initial PVS did not have any problems for a long period. However, EPVS placement was performed because characteristics of ascites were bloody and chylous.

**Table 1:** Patient characteristics for 10 patients who received EPVS

**No**.	age	sex	primary disease	nature of ascites	PS	indication for EPVS	EPVS duration, days	survival duration, days	outcome	AEs
1	22	F	synovial sarcoma	serous	3	poor PS	6	6	not effective	renal failure**
2	64	M	mesothelioma	serous	1	potential PVS dysfunction	3	133	effective*	none
3	46	M	lymphoma	serous	4	anasarca, poor PS	14	23	effective*	pulmonary edema**, diarrhea
4	62	F	ovary cancer	bloody	1	history of PVS occlusion	4	200	effective*	none
5	49	F	colon cancer	bloody	1	anasarca, potential PVS dysfunction	4	77	effective*	none
6	57	F	mesothelioma	serous	1	potential PVS dysfunction	16	133	effective*	none
7	56	F	breast cancer	chylous	1	potential PVS dysfunction	3	114	effective*	none
8	48	F	lung cancer	chylous	1	anasarca, history of PVS occlusion	28	28	effective	diarrhea, anemia
9	50	F	cholangiocarcinoma	chylous	4	anasarca, poor PS	24	24	effective	diarrhea
10	69	M	chondrosarcoma	serous	4	poor PS	2	2	not effective	renal failure**

median	52.3						10.4	74.0		

The EPVS system was removed and exchanged to conventional PVS on the same day.

PS: Eastern Cooperative Oncology Group Performance Status

EPVS: externalized peritoneovenous shunt

PVS: peritoneovenous shunt, AEs: adverse events

*: EPVS was exchanged to conventional PVS

**: major adverse event

Technical success was obtained in all patients without any major AEs that were associated with the procedure. EPVS placement provided clinical efficacy in 8 (80%) patients, and the procedure was effective for abdominal distention in 8 patients, anorexia in 5 patients, and nausea/vomiting in 4 patients (with overlap). The largest abdominal girth was resolved in all patients. However, given the low sample size, we were unable to perform a statistical analysis to test for a correlation between hematological parameters, abdominal girth, and diuresis, before or after EPVS placement (Table 2).

**Table 2:** Hematological parameters, abdominal girth, and diuresis, before and after EPVS placement

	Preoperative Period	3-POD	7-POD	14-POD
Cr, mg/dL	1.1 (0.6)	1.2 (0.7)	1.1 (0.8)	1.1 (0.9)
BUN, mg/dL	21.2 (10.6)	22.6 (13.7)	23.5 (13.7)	27.8 (15.8)
Albumin, g/dL	2.3 (0.3)	2.4 (0.4)	2.6 (0.5)	2.5 (0.6)
PT, %	70.1 (13.8)	60.8 (12.5)	68.6 (13.10)	67.6 (10.4)
Platelets, 10^3 ^cells/mm^3^	35.8 (9.1)	26.7 (10.6)	25.3 (8.7)	29.3 (6.8)
Largest abdominal girth, cm	87.5 (9.6)	84.1 (10.6)	81.7 (5.1)	80.2 (4.8)
Diuresis, 24 h, mL	978 (466)	1723 (560)	1612 (416)	1334 (506)

Data are presented as mean (SD).

POD: postoperative day

PT: prothrombin time

The median duration of EPVS placement was 10.4 days (range, 2-28 days). The EPVS was exchanged to conventional PVS sequentially in 6 patients (60%), since the initial EPVS placements resulted in improvement of subjective symptoms without major AEs, and the patients' life expectancies were suspected to be more than a month. In these 6 patients, the EPVS system was removed and exchanged to conventional PVS on the same day. There were no cases of EPVS and PVS dysfunction on the follow-up period. Patency period of EPVS and PVS were 10.4 days (range 2-28 days) and 106 days (9-196 days).

The median survival duration was 74.0 days (range, 2-200 days). The survival durations of the patients with poor PS (PS 3 or 4; n = 4) were less than 1 month (range, 2-24 days).

Three patients (30%) had major AEs. Acute death occurred within 7 days after EPVS placement in 2 patients (No. 1 and No.10) because of renal failure which had been occurred by hypovolemia since preoperative days. Before EPVS placement, these patients had renal dysfunction and low serum albumin levels. As one patient (No. 3) experienced pulmonary edema just after the EPVS placement, we clumped the catheter to avoid hyperperfusion on the 3^rd ^postoperative day until the pulmonary edema was resolved on the 5^th ^postoperative day.

Minor AEs were observed at 30% (diarrhea in three patients and anemia in one patient) and were resolved conservatively.

## Discussion

Our results suggest that EPVS placement for refractory ascites is effective and can be adopted even for patients who were not suitable for a conventional PVS implantation because of anasarca, poor PS, potential PVS dysfunction, or history of PVS occlusion. However, EPVS placement resulted in a 20% procedure-related early death. We suggest that careful assessment of the procedure-related risks and close monitoring after EPVS implantation are essential.

Malignant ascites in patients with advanced cancer is often resistant to treatment. Troublesome symptoms from ascites result in progressive deterioration of the patients' QOL. Diuretics and paracentesis have been traditionally employed to relieve the symptoms associated with ascites. However, their use has been inconsistent among physicians. It is sometimes difficult for patients or caregivers to bring patients to the place where paracentesis is performed. Repeated paracentesis requires frequent trips to the hospital with risks of hypovolemia, hypotension, and hypoproteinemia from ascites removal [6].

Although paracentesis can provide immediate relief, the effects may be temporary, and complications, such as bleeding, hypotension, secondary peritonitis, and loss of protein and electrolytes, may occur [13,14]. Since the first report by LeVeen et al. [1], the Denver PVS has been considered as one of the most common procedures used to treat intractable malignant ascites, in which other conservative medical therapies may not be effective [1-7].

Although PVS is simple and an about 70% clinical effectiveness is expected, AEs, such as pulmonary edema, pulmonary arterial embolism, and disseminated intravascular coagulation (DIC), following PVS implantation may frequently occur. In addition, shunt replacement (removal) is often warranted due to shunt infection or occlusion of the system [3-7]. Efficacy of PVS would not be superior to that of paracentesis in short period, therefore PVS implantation has been considered to be a contraindication in patients with malignant ascites due to gastrointestinal malignant tumors, and a shunt should only be used when the life expectancy of the patients expected to derive a benefit from it is more than 2 or 3 months ([3-5,15]).

Bieligk et al. [7] reported that preoperative impaired renal function was a predictive factor of poor prognosis after PVS insertion. Thus, careful consideration should be taken in deciding on placement in patients with insufficient urine volume, who may be unable to tolerate the rapid increase in plasma volume immediately after PVS insertion [5,7]. In fact, in our study, early death occurred within 7 days after EPVS placement in 2 patients because of progressive renal failure. Before EPVS placement, these patients had renal dysfunction and low serum albumin levels. After EPVS placement, ascites volume was decreased in these patients, although they had impaired production of sufficient urine volume. A possible explanation is that the low colloid osmotic pressure of these conditions may lead to extravascular transudation of water, resulting in renal failure due to hypovolemia [5,7,16,17].

Our EPVS procedure has some advantages over conventional (PVS) implantation. Not only EPVS is placement technically simpler, but EPVS may be less invasive for patients. A subcutaneous long tunnel is not need for EPVS. We can easily maintain the system, and the flow control is easily performed at any time. This is a very important advantage of EPVS placement, since PVS insertion may result in rapid changes in circulatory dynamics, as well as rapid introduction of various agents present in ascites into the circulation. For example, when severe complications, such as pulmonary edema or dyspnea, occur, we can readily occlude the shunt system by clamping the catheter. Conversely, pumping the chamber is easily performed. Thus, we can educate the patients and their family members on how to control the flow. It is easy to explain the mechanism of the PVS to patients and their families. Besides device maintenance, replacement and removal of the EPVS system are also far easier than conventional PVS [6,18]. We suspect that an EPVS placement can also be a preparatory step for standard conventional PVS implantation. In our series, in six patients, EPVS has been successfully exchanged to a conventional PVS system.

The development of a PVS that can be turned on or off would be a useful development, even though this would not really get around the anasarca problem. If a flow control valve could be incorporated into the circuit (like a ventriculoperitoneal shunt for hydrocephalus), this might solve some of the problems that can occur with PVS implantation.

There are a number of possible limitations with EPVS implantation. First, there are no consistent preoperative indicators associated with poor patient survival after EPVS placement, and it is difficult to know which patients will achieve a high rate of palliation with a low morbidity and mortality rate [3-6]. In cases of anasarca, it may be adequate to exchange EPVS so that abdominal distension will resolve. However, in other cases, it is difficult to seize a favorable occasion to convert from EPVS to conventional PVS. In our small cohort, a conversion to PVS depends upon the condition of the patients, and we could not but have tentative period or factors to decide when EPVS is exchanged to PVS. An improvement in nutritional status can improve the chance of a successful conversion to PVS. However, it is not easy to improve nutritional status in a patient at the end of life. We suspect the success of the conversion can occur if the major AEs are avoided after EPVS placement because EPVS placement is a temporary system that is used temporarily in place of conventional PVS. Previous studies have emphasized meticulous postprocedural management for 48 hours in order to detect major AEs [4,19]. Therefore, we suspect that increased attention should be given for more than 48 hours after the procedure. Second, EPVS placement may have a higher risk for system infection and migration, although we did not have any such cases in our series. However, the duration of the EPVS placements in this study is considerably short to define infection risks for any tunneled catheter, such as that used for pleural effusion, dialysis, central lines, etc. [6,18]. Third, there is an economical issue: when patients were able to get the favorable clinical course associated with EPVS placement, they had to pay for another Denver shunt kit in order to convert the EPVS system to PVS.

Additional limitations of the study include the following: the study was retrospectively performed, there were a small number of patients, and the follow-up period was very short. No consensus on how to evaluate the efficacy of PVS or EPVS has been established [20,21]. Our subjective procedure for determining the efficacy 7 days after EPVS insertion is controversial. Additionally, if ascites was removed with the PVS implantation procedure itself, it can palliate symptoms separately from an effect of the EPVS. We evaluated the improvement of anasarca as a decrease of abdominal girth, and there was no weight data in all patients. No consensus on how to evaluate anasarca in patients with malignant ascites. Abdominal girth may not an appropriate index of anasarca because abdominal girth may also represent decrease of ascites after PVS insertion.

A previous study recommended measuring body weight in order to evaluate PVS efficacy, but it may be occasionally difficult to measure body weight in patients at the end of life. In addition, patients with advanced malignancies may deteriorate rapidly due to the primary disease and other pathophysiologies that further confound evaluation. We attempted to minimize fluid overload. However, flow control is a regular and needed issue for percutaneous shunts, and it is difficult to assess circulatory dynamics [10]. In future studies, we need to assemble a larger cohort that we will follow over a longer term in order to evaluate efficacy and complications.

## Conclusion

In conclusion, EPVS placement may be an option method for patients with malignant ascites, who may not be appropriate for conventional PVS implantations. Our preliminary experience encourages further studies into the efficacy of EPVS placement.

## Competing interests

The authors declare that they have no competing interests.

## Authors' contributions

HT reviewed relevant literature and drafted the manuscript. All authors provided clinical expertise and participated in drafting the manuscript. And all authors read and approved the final manuscript.
